# Description of Catenibacterium mitsuokai subsp. tridentinum subsp. nov., an anaerobic bacterium isolated from human faeces, and emended description of C. mitsuokai

**DOI:** 10.1099/ijsem.0.006798

**Published:** 2025-05-30

**Authors:** Liviana Ricci, Marta Selma-Royo, Davide Golzato, Charlotte Servais, Amir Nabinejad, Paolo Marchi, Michal Punčochář, Francesco Trenti, Mar Garcia-Aloy, Federica Armanini, Roberta Marconi, Francesco Asnicar, Federica Pinto, Graziano Guella, Sabrina Tamburini, Nicola Segata

**Affiliations:** 1 Department of CIBIO, University of Trento, Trento, Italy; 2 IEO, European Institute of Oncology IRCCS, Milan, Italy; 3 Department of Physics, University of Trento, Trento, Italy; 4 Metabolomics Unit, Research and Innovation Centre, Fondazione Edmund Mach, San Michele All’Adige, Trento, Italy; 5 Department of Molecular Sciences and Nanosystems, Ca’ Foscari University, Venice, Italy; 6 Department of Twins Research and Genetic Epidemiology, King's College London, London, UK

**Keywords:** human gut microbiota, non-urbanized lifestyle, *tridentinum *subspecies

## Abstract

A recent metagenomic survey has revealed an unknown bacterial clade within the *Catenibacterium mitsuokai* species to be significantly more prevalent in non-urbanized populations, compared to urbanized ones. We isolated and characterized a strain of this clade from the stool of a healthy adult volunteer. Strain CMD8551^T^ is strictly anaerobic, appears as long chains of Gram-positive rods and produces acetate in the presence of glucose. The lipidomic profile showed a higher proportion of saturated lipid species amongst the detected phospholipids. The whole genome is 2,320,430 bp long and has a G+C content of 33.7 mol% with 2,239 CDSs. A phylogenetic analysis comparing the sequences of the strain CMD8551^T^ with publicly available reference genomes from the *Catenibacterium* genus revealed that the CMD8551^T^ isolate, together with other isolate genomes, forms a distinct subspecies of *C. mitsuokai* and has an average nucleotide identity lower than 94% with respect to the previously described *C. mitsuokai* subsp. *mitsuokai*. Given the phenotypic, chemotaxonomic and phylogenetic characteristics of the newly isolated CMD8551^T^ (=DSM 118469^T^=LMG 33725^T^=CIP 112509^T^) that clearly differ from those of the *C. mitsuokai* subsp. *mitsuokai* type strain RCA14-39^T^, we propose it as the type strain of a novel subspecies of *C. mitsuokai*, with the name *C. mitsuokai* subsp. *tridentinum* subsp. nov.

## Introduction

The human microbiota is involved in several physiological processes [1,2] and is essential in maintaining host health [3]. Despite the advances in uncovering the bacterial diversity of the human gut microbiota driven by cultivation-free sequencing technologies [4], the proportion of microbes with cultivated and phenotypically described members is still scarce. Sustained effort in characterizing and classifying currently undescribed taxa of the human microbiome remains crucial to unravel the complex structure and function of the human microbiome.

The *Catenibacterium* genus is one of the clades with only a partially uncovered diversity. This genus was described over two decades ago as *Eubacterium*-like bacteria but with distinct genetic and metabolic characteristics [5]. The genus belongs to the family *Coprobacillaceae* (*Erysipelotrichales* order) with only one known species, *Catenibacterium mitsuokai*, that was isolated from human faeces from Papua New Guinea highlanders.

Here, we describe *C. mitsuokai* subsp. *tridentinum* subsp. nov., based on a strain recovered from a human stool sample of a healthy adult. Recent metagenomic surveys were able to reconstruct 882 genomes assigned to this previously undescribed subspecies and pinpointed this distinct subspecies to be significantly more prevalent in the faecal microbiota of populations characterized by a non-urbanized lifestyle (63% average prevalence in the 18 non-urbanized datasets surveyed) compared to cohorts within an urbanized setting (10% [6]). The *C. mitsuokai* subsp. *tridentinum* subspecies, and not the subsp. *mitsuokai*, is also highly transmitted amongst adult individuals residing in the same household according to previous studies [7].

## Methodology

### Isolation and ecology

*C. mitsuokai* subsp. *tridentinum s*train CMD8551^T^ was isolated from a fresh human stool sample from a healthy male adult resident in Trento (Italy) (protocol no. 2021-007 approved by the Ethical Committee of the University of Trento), after obtaining the volunteer’s informed consent to participate in the study [8]. In the same experiment and under the same conditions, an isolate of *C. mitsuokai* subsp. *mitsuokai* (strain CM85Y_71) was also obtained. In brief, the fresh stool was 10% (w/v) diluted in pre-reduced PBS, vortex-homogenized and serially diluted tenfold, under anaerobic conditions. One faecal dilution (10^−5^) was then spread (100 µl) onto modified anaerobic chopped meat agar (Oxoid), supplemented with 4% defibrinated sheep blood (Microbiol Diagnostics), 0.2% carbon sources including pectin from apple (Sigma), arabinoxylan from wheat (Megazyme), d-glucose (Sigma), d-maltose (Fluka AG), d-cellobiose (Cayman Chemical), soluble starch from potato (BD), 2.5 g l^−1^ yeast extract (Fluka AG), 0.005% vitamin K1 (Alfa Aesar) and 5 mg l^−1^ hemin (Thermo Fisher), for 3 days at 37 °C under anaerobic conditions. Single colonies were inoculated in modified chopped meat broth and incubated for 48 h. Liquid cultures were then used to produce bacterial stocks in 20% (v/v) glycerol and kept at −80 °C and to taxonomically identify the isolates.

### Bacterial identification

Genomic DNA was extracted from the bacterial culture using the Wizard^®^ Genomic DNA Purification Kit (Promega) and subsequently purified with the QIAquick PCR Purification Kit (Qiagen). The 16S rRNA gene was amplified using the universal bacterial primers (8F 5′-AGAGTTTGATCCTGGCTCAG-3′ [9] and 1492R 5′-GGTTACCTTGTTACGACTT-3′ [10]) and Sanger sequenced (Eurofins Genomics) for an initial taxonomic description. blastn [11] alignment (16S_ribosomal_RNA database) identified *C. mitsuokai* 16S rRNA gene (NR_027526.1) as the closest match, with 97.72% identity (97% query coverage). A further sequence mapping analysis against the MetaRef database (version June 2023) [4,6] matched the 16S rRNA gene sequence of an unknown clade within the *Catenibacterium* genus.

### Whole-genome sequencing and genome characterization

Whole-genome sequences were produced as previously described [8] and are publicly available on NCBI (study project accession ID PRJNA939950, assembly accession ID GCA_029675045.1), together with the 16S rRNA gene sequence (PP999749.1). Taxonomic assignment and phylogenetic analyses were performed using PhyloPhlAn version 3.1 [12], updating the results reported previously [8]. Pairwise average nucleotide identity (ANI) between genomes was computed using skANI [13]. Similar phylogenetic analyses were performed for the isolate *C. mitsuokai* subsp. *mitsuokai* CM85Y_71, which showed an ANI of 97.8% to the type strain *C. mitsuokai* subsp. *mitsuokai* RCA14-39^T^.

To have a better understanding of the specific genome features of *C. mitsuokai* subsp. *tridentinum*, we generated a phylogenetic tree of strain CMD8551^T^ and related taxa within the *Catenibacterium* genus with available reference genomes obtained from sequencing of isolates. A more extensive phylogeny, including our isolate genome as well as all reference and metagenomic assembled genomes (MAGs) within the genus available in the ChocoPhlAnSGB database (Jan 25 version), was included in Fig. S1, available in the online Supplementary Material [6]. A total of 1,141 reference and high-quality MAGs (>90% completeness, <5% contamination) were considered, including *C. mitsuokai* subsp. *mitsuokai* (n_MAG_=188, n_REF_=5), *C. mitsuokai* subsp. *tridentinum* (n_MAG_=897, n_REF_=11) and other genomes present in the *Catenibacterium* genus (n_MAG_=40). Identification of the 1,164 core genes (>60% coreness) [14] in these genomes allowed us to generate a phylogeny using the PhyloPhlAn pipeline.

To explore and compare the functional capabilities of *C. mitsuokai* subsp. *mitsuokai* and *C. mitsuokai* subsp. *tridentinum*, we considered the annotations obtained using Prokka (version 1.14) of all the genomes assigned to the two subspecies in the ChocoPhlAnSGB database (Jun 23 version; total *n*=911 genomes, including ref. genomes and high-quality MAGs), which were further functionally characterized by UniRef90/50 mapping (version 2019–06) [15]. The genome profiles according to the UniRef90 presence or absence patterns from all genomes were then used to build a matrix to compare the pangenomes of the two subspecies. Genes that had an overall prevalence below 5% across all the genomes were removed from the pangenome analysis, together with genes that could not be assigned to any of the UniRef90 gene families.

The translated ORFs of the 911 genomes were then annotated for KEGG orthologues (KOs) using the pipeline *Microbeannotator* [16], which uses kofam scan (https://github.com/takaram/kofam_scan) to annotate the ORFs. The Kofam database version 23-04-01 (based on KEGG database 106.0) was used. KOs were associated with their functional category of belonging using the KEGG BRITE hierarchy. Complete annotations of *C. mitsuokai* subsp. *mitsuokai* and *C. mitsuokai* subsp. *tridentinum* pangenomes are provided in Tables S1 and S2. We then compared the frequency of core genes (90% or higher prevalence within genomes of the same species) associated with different KO functional categories across genomes of the two subspecies. Sporulation potential was genomically explored *in silico* by identification of the KOs associated with sporulation in the pangenomes of the two subspecies (Table S3).

### Bacterial cultivation and phenotypic analysis

*C. mitsuokai* subsp. *tridentinum* CMD8551^T^ was routinely maintained by overnight growth in Yeast Casitone Fatty Acids (YCFA) medium [17] supplemented with glucose, sucrose and maltose (YCFA-GSM) or Peptone Yeast Glucose (PYG) broths at 37 °C, anaerobically (5% H_2_, 10% CO_2_, balance N_2_). All media were prepared using oxygen-free carbon dioxide. Overnight bacterial cultures were used for Gram staining following standard procedures. Cellular morphology was observed using scanning electron microscopy (SEM) on bacteria grown in YCFA-GSM broth. Sample preparation for SEM was performed as previously described [18]. SEM images were analysed by the Fiji software [19] to detect bacterial cells using the MicrobeJ plugin. Then, length and width were measured in 70 cells detected in five different imaged areas.

Carbohydrate utilization and enzymatic activity profiles were determined using the standardized API strips tests 20A and Rapid ID 32A (bioMérieux), in duplicate and as per manufacturer’s instructions. The API20A failed to provide robust positive reactions for CMD8551^T^, so carbon substrate utilization of the isolate was assessed in triplicate in 96-well plates using basal YCFA supplemented with 0.5% single substrates such as d-glucose (Sigma), d-sucrose (Thermo Fisher), d-maltose (Sigma), d-cellobiose (Cayman Chemical), l-arabinose (Sigma), d-xylose (Sigma), d-sorbitol (Sigma), d-mannitol (Sigma), pectin from apple (Sigma), inulin from chicory (Sigma), soluble starch (BD) and larch wood arabinogalactan (Sigma) (Table S4). Growth was monitored by changes in OD at 600 nm (OD_600_), during 24 h incubation in the anaerobic atmosphere.

Motility was assessed by culturing CMD8551^T^ in 0.2% agar PYG slants [20] at 37 °C; results were observed 48 h after inoculation. Isolates from our bacterial collection [21], previously assigned to the species *Enterococcus faecalis* and *Escherichia coli*, were used as negative and positive controls, respectively.

Spore formation was assessed following alcohol treatment, as previously described [22], using *Bacillus subtilis* strain 168 DSM402 and *E. coli* (from our bacterial collection [21]) as positive and negative controls, respectively. Catenibacteria were incubated under anaerobic conditions, whilst the control species were aerobically cultured at 37 °C for 24 h. All the experiments were performed in triplicates. The purchased live culture of *C. mitsuokai* subsp. *mitsuokai* (RCA14-39^T^ type strain, DSM15897^T^) was included in all the phenotypic analyses for comparison purposes.

### Susceptibility to antibiotics and bile acids and to environmental temperature, pH and oxygen

Susceptibility to antibiotics of strain CMD8551^T^ and strain RCA14-39^T^ was tested by plating 200 µl of bacterial overnight liquid culture on PYG agar plates (pH 7) using the disc diffusion method [23]. The following antibiotics and quantities were tested: amoxicillin, 20 µg (BioRad); chloramphenicol, 30 µg (BioRad); ciprofloxacin, 5 µg (BioRad); erythromycin, 15 µg (BioRad); gentamicin, 10 µg (BioRad); kanamycin, 30 µg (BioRad); neomycin, 30 µg (Mast Group); tetracycline, 30 µg (BioRad); penicillin, 6 µg (BioRad); streptomycin, 10 µg (BioRad); vancomycin, 5 µg (BioRad). The inhibition area was measured after a 48 h incubation under anaerobic conditions.

Sensitivity to bile extract (0–2.5%, w/v) (Sigma-Aldrich), temperature (28, 37 and 44 °C) and pH (pH range 2–8) were tested in PYG broth contained in Hungate tubes at 37 °C during 48 h. Bacterial growth was assessed by spectrophotometrically measuring OD_600_.

To investigate oxygen susceptibility, overnight PYG broth cultures of strain CMD8551^T^ and strain RCA14-39^T^ were diluted to an OD_600_ of 0.1 (considered as T_0_), and 1 ml of bacterial suspension was inoculated in a 24-well plate (3524 Corning^®^). Cultures were either incubated anaerobically for 24 h (control condition) or aerobically for 1 or 2 h and then anaerobically for the remaining time until 24 h from primary inoculation (1 and 2 h air conditions, respectively). After 24 h (at T_24_), bacterial growth was monitored by measuring changes in OD_600_. For each susceptibility assay, two-way ANOVA tests were performed including bacterial species and single environmental conditions as factors, using GraphPad v. 8.4.3 (GraphPad Software, Boston, MA, USA, www.graphpad.com). Sidak’s multiple comparisons post-hoc test was then used to identify significant differences (adjusted *P* value<0.05) in growth between species, for each condition. Results are shown in Fig. S2 and Table S5.

### Production of volatile fatty acids

Production of short-chain fatty acids (FAs) (SCFAs) was evaluated via GC–MS analysis of bacterial culture supernatants. These were obtained from 4,000 ***g***, 10 min centrifugation of overnight cultures in PYG broth of *C. mitsuokai* subsp. *tridentinum* CMD8551^T^ and *C. mitsuokai* subsp. *mitsuokai* RCA14-39^T^. SCFA extraction and GC–MS analysis were performed as previously described [24].

### Cellular lipid description

The cellular lipid composition of strain CMD8551^T^ was characterized at the Metabolomics Unit of the Fondazione Edmund Mach (San Michele all’Adige, TN, Italy) using LC-MS as outlined previously [25]. For this analysis, *C. mitsuokai* subsp. *mitsuokai* strain CM85Y_71, isolated as part of this study, was utilized as a comparison. Overnight YCFA-GSM broth cultures were pelleted by centrifugation (4,000 r.p.m., 5 min), and cells were washed twice with PBS. Total lipids were extracted and purified [26] and analysed using the Acquity UPLC CSH-C18 column (2.1×100 mm, 1.7 µm; Waters, Milford, MA, USA). The chromatographic system (Dionex Ultimate 3000 UHPLC) was coupled with a high-resolution Orbitrap LTQ-XL mass spectrometer (Thermo Fisher, Bremen, Germany), equipped with an electrospray ionization source. The Orbitrap was operated both in positive and negative ionization and data-dependent acquisition modes. Analyses were performed with four technical replicates per sample, comprising two different volumes (2.5 and 5 µl for strain CM85Y_71 and 5 and 10 µl for strain CMD8551^T^, based on sample extract concentrations of 10 and 5 mg ml^−1^, respectively) injected in duplicate. Lipids were identified based on their exact mass, retention time behaviour, fragmentation patterns and the type of generated ion according to the polarity mode. The annotation criteria followed the approach described in [25]. Results were presented as % of each lipid category compared to the total sum of the corresponding class. Detailed data distribution of the chromatographic peak areas (corrected for injection volume and sample concentration) across the two subspecies was also included (Fig. S3).

## Results

### Phylogeny

The comprehensive phylogeny including both publicly available reference genomes as well as high quality MAGs (Fig. S1) revealed the *Catenibacterium* genus to comprise five species-level clusters, the largest of which being the type species of the genus, *C. mitsuokai*, in addition to four undescribed species-level clusters composed solely of MAGs.

**Fig. 1.**
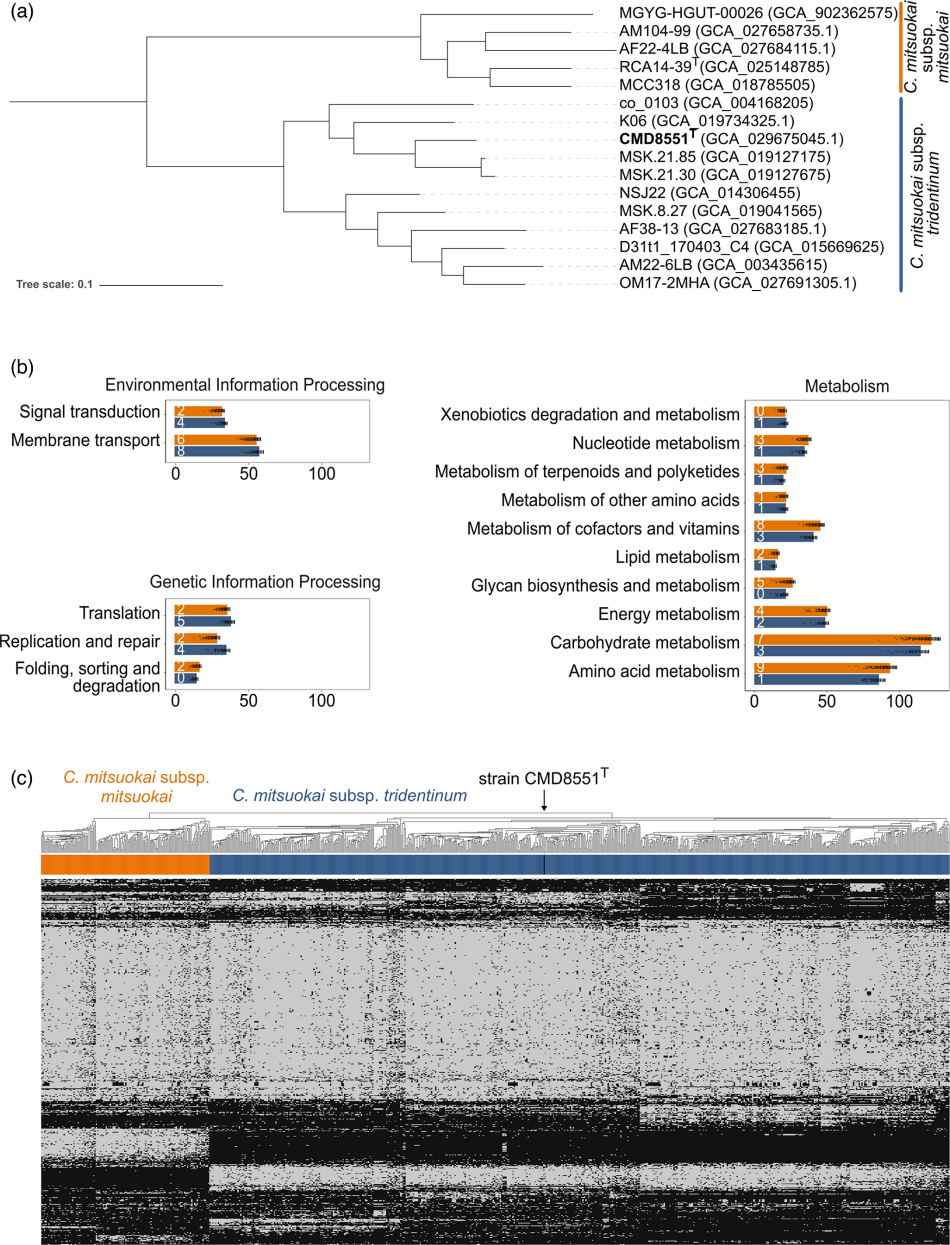
Genomic characterization of *C. mitsuokai* subsp. *tridentinum* and *C. mitsuokai* subsp. *mitsuokai*. (**a**) Phylogenetic tree of the *Catenibacterium* genus including all reference genomes available in the ChocoPhlAnSGB database (Jan 25 version). (**b**) Comparison of the frequency of core genes (90% of prevalence within genomes of the same subspecies) associated with different KEGG orthologue (KO) functional categories across genomes of *C. mitsuokai* subsp. *tridentinum* and subsp. *mitsuokai* (ChocoPhlAnSGB vJun23; *n*=911 genomes). Only functional categories whose difference was statistically significant (two-sided Wilcoxon test, *P* value <0.05) between the two species were included in the figure. The number inside each barplot reports the number of KOs that are exclusive to a subspecies. (**c**) The heatmap plot shows UniRef90 profiles (rows) across genomes (columns) composing the pangenome matrix hierarchically clustered (Jaccard distance) (ChocoPhlAnSGB vJun23). Grey and black colours indicate the presence and absence of genes, respectively.

A simpler phylogeny, including only the 16 genomes from isolates taxonomically assigned to the *Catenibacterium* genus and available in public databases, highlighted the presence of two differentiated clades within the *C. mitsuokai* species (Fig. 1a). The one comprising the previously described *C. mitsuokai* subsp. *mitsuokai* strain RCA14-39^T^ (GCA_025148785.1) designated as the type strain (and used in this study for comparison purposes) [5], with an overall intra-subspecies ANI ranging from a minimum of 96.26% to a maximum of 98.04%. In contrast, strain CMD8551^T^ formed a distinct subtree from that of *C. mitsuokai* subsp. *mitsuokai* (inter-subspecies ANI ranging from 93.78 to 94.46%), supporting the designation of the novel *tridentinum* subspecies characterized in this work. This was the largest subspecies, comprising strain NSJ-22 (formerly named *Catenibacterium faecis* [27]), four publicly available genomes previously assigned to *C. mitsuokai* in addition to five with undefined taxonomy.

As previously reported [5], several strains of *Kandleria vitulina* appeared as the closest known species to the *Catenibacterium* genus, in particular showing an ANI of 71.16% (min–max: 77.937–78.303%) with the CMD8551^T^ genome.

### Genome features

Genome statistics of strain CMD8551^T^ (Table 1) were analysed in comparison with *C. mitsuokai* subsp. *mitsuokai* RCA14-39^T^. The two strains showed an equal number of tRNA (*n*=76), whilst RCA14-39^T^ presented more rRNA (*n*=27 vs *n*=10) and genes (*n*=2696 vs *n*=2326), and higher G+C content (34.68 mol% vs 33.7 mol%) than CMD8551^T^. However, the observed differences in genome features such as the number of rRNA may be due to incomplete assembly of genomes and the known issues in assembling multiple copies of highly homologous gene sequences.

**Table 1.** Characteristics of *C. mitsuokai* subsp. *tridentinum* CMD8551^T^ and *C. mitsuokai* subsp. *mitsuokai* RCA14-39^T^ genomes

Attribute	CMD8551^T^	RCA14-39^T^
Size (bp)	2,320,430	2,650,196
G+C content (mol%)	33.7	34.68
CDSs	2,239	2,592
rRNA genes	10	27
tRNA genes	76	76

Predictive analysis of encoded functional capabilities, performed using the UniRef90 and KEGG databases to annotate the genomes, revealed that whilst the subspecies shared several genes for all the KEGG categories, subsp. *tridentinum* showed a higher number of genes related to the metabolism of carbohydrates, amino acids, cofactors and vitamins (Fig. 1b), and, more generally, substantial genomic differences in the overall repertoire of core genes (coreness of 95%), compared to subsp. *mitsuokai* (Fig. 1c).

Sporulation potential was explored by identification of KOs associated with spore formation in the pangenomes of the two subspecies. Single genes involved in sporulation were identified in subsp. *tridentinum*, including spo0A (K07699) as well as additional genes involved in spore formation in subsequent stages (i.e. K06413, stage V sporulation protein K that was found in both subspecies); however, additional genes determinant to initiate sporulation via spo0A were not detected in subsp. *tridentinum*, suggesting the presence of incomplete sporulation pathways (Table S3).

### Bacterial morphology and metabolic capabilities

**Fig. 2.**
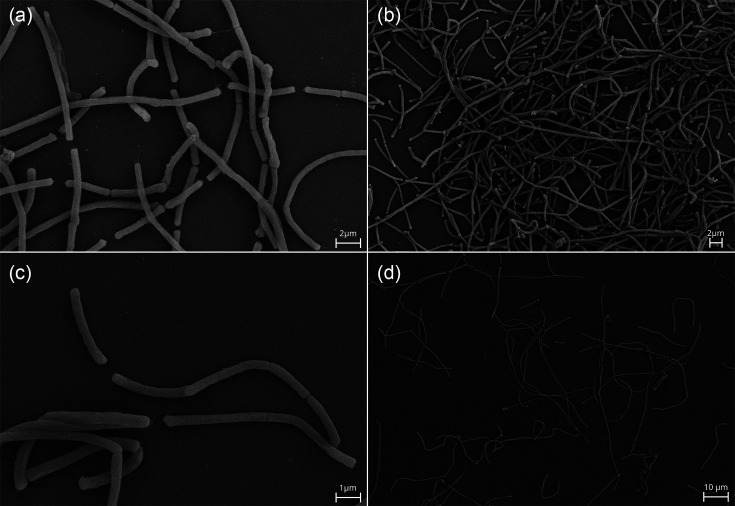
Electron microscopy images of *C. mitsuokai* subsp. *tridentinum* (**a, b**) and *C. mitsuokai* subsp. *mitsuokai* (**c, d**).

Strain CMD8551^T^ is Gram-positive and grows in chains of rods (Fig. 2). The cell is ~3.6±1.1 µm long (min–max: 2.2–6.24) and shows a diameter of 0.51±0.06 µm (min–max: 0.41–0.7). Using electron microscopy, both *tridentinum* and *mitsuokai* subspecies appear to have a similar cell morphology with the rods organized in long chains of variable length (Fig. 2). Whilst, in the * C. mitsuokai* sp. description [5], the cell was identified as 1.2–2.0 µm long, our analysis showed no differences between the cellular length of the two subspecies (subsp. *mitsuokai* length=3.78±0.78; T-test, *P*=0.282).

Strain CMD8551^T^ is non-motile, obligately anaerobic and does not form spores. Its growth is stimulated by the supplementation of glucose, cellobiose, sucrose and maltose to YCFA broth at 37 °C, but it also exhibits robust growth in PYG media. After 48 h of incubation, CMD8551^T^ colonies are punctiform and lightly cream-coloured.

Metabolic capabilities of CMD8551^T^ were further investigated utilizing the API Rapid ID 32A (bioMérieux) and showed a very similar pattern between the two species with the exception of the catalysis of l-leucyl-l-glycine-b-naphthylamide that was identified only in *C. mitsuokai* (Table S4).

The detected primary metabolite produced by CMD8551^T^ in PYG medium is acetate.

### Susceptibility to antibiotics, bile extracts, environmental temperature, pH and oxygen

Both *C. mitsuokai* subsp. *mitsuokai* RCA14-39^T^ and *C. mitsuokai* subsp. *tridentinum* CMD8551^T^ showed optimal growth at 37 °C, with the latter displaying significantly higher ODs at 44 °C compared to RCA14-39^T^ (*P*=0.017, T-test) (Fig. S2 and Table S5). Generally, strain CMD8551^T^ showed similar susceptibility to tested pH, bile extracts and oxygen stressors, to the subsp. *mitsuokai* type strain. In particular, the growth of both strains was impaired by all the assayed bile salt concentrations, compared to the control condition (*P*<0.001; no bile salt supplementation), with no significant differences between them (Fig. S2 and Table S5). Antibiotic susceptibility testing revealed an overall higher sensitivity of strain CMD8551^T^ compared to RCA14-39^T^, with significant differences between the two strains when exposed to gentamicin, erythromycin, tetracycline, ciprofloxacin, chloramphenicol, vancomycin and neomycin (Table 2). CMD8551^T^ was found to be resistant to streptomycin (10 µg) and kanamycin (30 µg) and displayed mild resistance to gentamicin (10 µg) and neomycin (30 µg) (Table 2).

**Table 2.** Antibiotic susceptibilities of *C. mitsuokai* subsp. *tridentinum* CMD8551^T^ and *C. mitsuokai* subsp. *mitsuokai* RCA14-39^T^

Antibiotic	CMD8551^T^	RCA14-39^T^	Adj. *P* value
Gentamicin (10 µg)	0.2 (0)	0.0 (0)	0.0041**
Streptomycin (10 µg)	0.0 (0)	0.0 (0)	>0.9999
Kanamycin (30 µg)	0.0 (0)	0.0 (0)	>0.9999
Erythromycin (15 µg)	1.0 (0)	0.8 (0)	0.0041**
Tetracycline (30 µg)	2.0 (0)	1.7 (0)	<0.0001****
Ciprofloxacin (5 µg)	0.5 (0)	0.9 (0.1)	<0.0001****
Penicillin (6 µg)	1.5 (0)	1.4 (0)	0.4155
Chloramphenicol (30 µg)	1.5 (0)	1.2 (0)	<0.0001****
Amoxicillin (20 µg)	2.0 (0)	1.9 (1.1)	0.4155
Vancomycin (5 µg)	0.9 (1.1)	0.7 (0.1)	0.0003***
Neomycin (30 µg)	0.3 (0)	0.1 (0)	0.0041**

The table shows the mean diameter (sd) of bacterial clearance around the antibiotic disc (cm) (*n*=2). Asterisks refer to significant differences in antibiotic susceptibility between subspecies that were assessed by two-way ANOVA (interaction *P* <0.0001), followed by Sidak’s multiple comparison test (**, *P* ≤ 0.01; ***, *P* ≤ 0.001; ****, *P* ≤ 0.0001).

With regards to environmental stressors, negligible growth was observed for both strains at a pH lower than 5 (60.74%±6.60 and 48.93%±4.18 for CMD8551^T^ and RCA14-39^T^, respectively; *P*=0.387). Whilst RCA14-39^T^ growth was not improved by a pH increment from 6 to 7 (increment of 1.43%±1.55), CMD8551^T^ growth increased at pH 7 by more than 50% compared to pH 6 (increment of 64.45%±12.48) (CMD8551^T^ vs RCA14-39^T^ growth increment at pH 7, *P*<0.001) (Fig. S2 and Table S5). Finally, both strains appeared to be anaerobic with non-significant differences in oxygen tolerance (Fig. S2 and Table S5).

### Cellular FAs

**Fig. 3.**
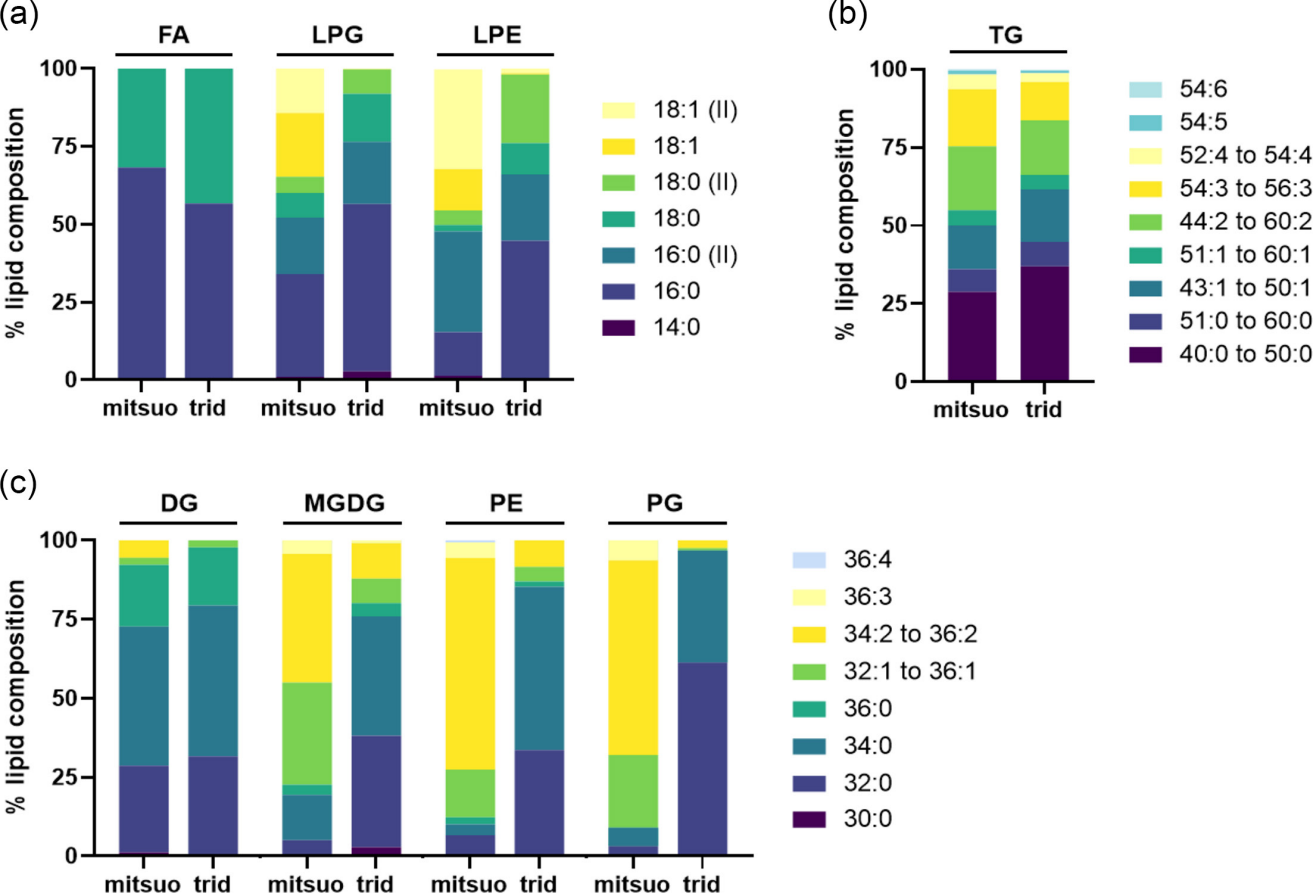
Cellular lipid composition of *C. mitsuokai* subsp. *tridentinum* CMD8551^T^ and *C. mitsuokai* subsp. *mitsuokai* CM85Y_71. Lipids are presented as % of each lipid category compared to the total sum of the corresponding class, classified according to the number of side chains (panels a, **b and c**) and colour-coded according to the number of unsaturations (dark colours for 0 or low number of unsaturations and yellow and light blue for higher number of unsaturations). Abbreviations: mitsuo, CM85Y_71; trid, CMD8551^T^; LPEs, lyso-phosphatidylethanolamines; LPGs, lyso-phosphatidylglycerols; TGs, triglycerides; DGs, diglycerides; MGDGs, monogalactosyl-diacylglycerols; PEs, phosphatidylethanolamines; PGs, phosphatidylglycerols.

The lipidome of analysed samples was composed of free FAs, lyso-phosphatidylethanolamines (LPEs), phosphatidylethanolamines (PEs), lyso-phosphatidylglycerols (LPGs), phosphatidylglycerols (PGs), mono-galactosyldiacylglycerols (MGDGs), diglycerides (DGs) and triglycerides (TGs). Striking differences were observed in cellular FA composition between *C. mitsuokai* subsp. *tridentinum* CMD8551^T^ and *C. mitsuokai* subsp. *mitsuokai* CM85Y_71. Indeed, whilst *mitsuokai* CM85Y_71 displayed more variety in terms of unsaturations, *tridentinum* CMD8551^T^ included mainly saturated fats (Fig. 3), particularly for LPEs, PEs, LPGs, PGs and MGDGs. For instance, whilst LPE 18 : 1, as well as PE (34 : 1, 36 : 2, 36 : 3, 36 : 4) and MGDG 36 : 1, was highly abundant in strain CM85Y_71, they were limitedly or not detected in *tridentinum* CMD8551^T^ (Fig. S3). In contrast, smaller differences in lipid composition in terms of both the length of the acyl chain and the degree of unsaturation were observed for FA, DG and TG.

## Conclusion

We describe a novel subspecies within the *C. mitsuokai* species, for which we propose the name *C. mitsuokai* subsp. *tridentinum* and the newly isolated CMD8551^T^ as the type strain. In summary, the *in silico* whole-genome and pangenome analyses, as well as the cellular FA composition, and *in vitro* substrate utilization and antibiotic sensitivity tests performed in this study support the identification of two distinct subspecies of *C. mitsuokai* (Table 3). By doing so, we also propose a taxonomic reclassification of publicly available reference genomes belonging to the *C. mitsuokai* sp., which our ANI analysis found to be previously misassigned in NCBI (Fig. 1a).

**Table 3.** Genomic and phenotypic characteristics used to distinguish *C. mitsuokai* subsp. *tridentinum* CMD8551^T^ from *C. mitsuokai* subsp. *mitsuokai* RCA14-39^T^

	CMD8551^T^	RCA14-39^T^
rRNA	10	27
DNA G+C content (mol%)	33.7	34.68
CDSs	2,239	2,592
Leucyl-glycine arylamidase	−	+
Fermentation products*	A	A, B, L†
Acid production from:		
Maltose	+	−
pH range (optimum)	7–7.5	6–7
Temperature range (optimum)	37–40 °C	37 °C
Antibiotic resistance to	Streptomycin, kanamycin	Gentamicin, streptomycin, kanamycin

*A, acetate; B, butyrate; L, lactate.

†As described in [5].

### Protologues

#### Emended description of *Catenibacterium mitsuokai* [5]

*Catenibacterium mitsuokai* (mit.su.oˊkai. N.L. gen. n. mitsuokai of Mitsuoka, named after K. Mitsuoka, a Japanese microbiologist).

The following characteristics emended those reported in the original description of the *C. mitsuokai* species by Kageyama and Benno [5].

Cells are rod-shaped and occur in tangled chains. They are Gram-positive, obligately anaerobic, mesophilic, non-spore-forming, non-motile and fermentative. Strains ferment glucose, sucrose, cellobiose, mannose, raffinose and inulin but do not ferment sorbitol or mannitol. Some strains are negative for aesculin. The species includes two subspecies, *C. mitsuokai* subsp. *mitsuokai* and *C. mitsuokai* subsp. *tridentinum*, both isolated from human faeces. The five publicly available reference genomes of *C. mitsuokai* subsp. *mitsuokai* show a mean genome size of 2.49 Mb (min–max: 2.32–2.58 Mb) and a mean G+C content of 34.14 mol% (min–max: 33.75–35.20%), whilst the 11 reference genomes assigned to *C. mitsuokai* subsp. *tridentinum* display a mean genome size of 2.44 Mb (min–max: 2.28–2.65) and a mean G+C content of 34.05 mol% (min–max: 33.71–34.68%) (Fig. 1a). Encoded functional capabilities differentiating the type strains of the two subspp. are reported in Fig. 1b, and genome statistics are shown in Table 1. The type strain of *C. mitsuokai* and *C. mitsuokai* subsp. *mitsuokai* is RCA14-39^T^.

## Description of *Catenibacterium mitsuokai* subsp. *mitsuokai* subsp. nov.

*C. mitsuokai* subsp. *mitsuokai* (mit.su.oˊkai. N.L. gen. n. mitsuokai of Mitsuoka, named after K. Mitsuoka, a Japanese microbiologist) is mesophilic and obligately anaerobic. Cells grow in chains of rods, with a cell size of 0±4 µm×3.78±0.78 µm. It is non-motile and does not form spores. It can be cultivated in PYG for 2 days at 37 °C under anaerobic conditions, with an optimal pH range of 6–7. As previously described by Kageyama and Benno [5], cells produce acid from glucose, mannose, galactose, fructose, sucrose, maltose, cellobiose, lactose and salicin. Fermentation of glucose produces mainly acetate, butyrate and lactate. Starch hydrolysis is positive, whilst aesculin hydrolysis is negative. The cells do not produce gas, indole or hydrogen sulphide, and they do not reduce nitrate or liquefy gelatin. The cell wall contains A1c-type peptidoglycan with an (l-Ala)-d-Glu-m-Dpm peptide subunit. Its cellular lipidome comprises a diverse range of saturated and unsaturated fats. The type strain of *C. mitsuokai* subsp. *mitsuokai* is strain RCA14-39^T^ (JCM 10609^T^=CIP 106738^T^=DSM 15897^T^), isolated from human faeces.

The GenBank accession numbers for the 16S rRNA gene and the genome sequence of strain RCA14-39^T^ are AB030224 and GCA_000173795, respectively.

## Description of *Catenibacterium mitsuokai* subsp. *tridentinum* subsp. nov.

*Catenibacterium mitsuokai* subsp. *tridentinum* (tri.den.ti’num. L. neut. adj. *tridentinum*, pertaining to *Tridentum*, the Latin name of the city of Trento, Italy) is a Gram-positive subspecies belonging to the *C. mitsuokai* species that includes another known subspecies, *C. mitsuokai* subsp. *mitsuokai*. *C. mitsuokai* subsp. *tridentinum* is mesophilic and obligately anaerobic. Cells grow in long chains of rods that have a mean diameter of 0.5 µm and a length of 3.5 µm, approximately. It is non-motile and non-sporulating, as *C. mitsuokai* subsp. *mitsuokai*. It can be cultivated in PYG for 2 days at 37 °C in an anaerobic atmosphere; the optimal pH range is 7–7.5. Growth is enhanced by supplementation of maltose, sucrose, glucose, cellobiose and inulin. It produces acetate in the presence of glucose. The cellular lipidome mainly includes saturated fats, particularly for LPE, PE, LPG, PG and MGDG classes. The type strain of *C. mitsuokai* subsp. *tridentinum* is the strain CMD8551^T^ (=DSM 118469^T^=LMG 33725^T^=CIP 112509^T^) isolated from human faeces collected from a healthy volunteer.

## Supplementary material

10.1099/ijsem.0.006798 Supplementary Figures.

10.1099/ijsem.0.006798 Supplementary Tables.

## Abbreviations

ANI: average nucleotide identity
DGs: diglycerides
FAs: fatty acids
KO: KEGG orthologue
LPEs: lyso-phosphatidylethanolamines
LPGs: lyso-phosphatidylglycerols
MAGs: metagenomic assembled genomes
MGDGs: mono-galactosyldiacylglycerols
OD_600_: OD at 600 nm
PEs: phosphatidylethanolamines
PGs: phosphatidylglycerols
SCFAs: short-chain FAs
SEM: scanning electron microscopy
TGs: triglycerides
YCFA-GSM: YCFA supplemented with glucose, sucrose and maltose
