# Supplementary material for: Description of Catenibacterium mitsuokai subsp. tridentinum subsp. nov., an anaerobic bacterium isolated from human faeces, and emended description of C. mitsuokai
Source: Int J Syst Evol Microbiol. 2025 May 30;75(5):006798. doi: 10.1099/ijsem.0.006798 (PMC12125477; doi:10.1099/ijsem.0.006798)
Supplement: Supplementary Figures. [file ijsem-75-06798-s001.pdf]

Catenibacterium mitsuokai subsp. *tridentinum* subsp. nov., an anaerobic bacterium isolated from human faeces

Liviana Ricci[1#], Marta Selma-Royo[1#], Davide Golzato[1#], Charlotte Servais[1], Amir Nabinejad[2], Paolo Marchi[1], Michal Punčochář[1], Francesco Trenti[3], Mar Garcia-Aloy[4], Federica Armanini[1], Roberta Marconi[1], Francesco Asnicar[1], Federica Pinto[1], Graziano Guella[3], Sabrina Tamburini[2,5], Nicola Segata[1,2,6] *.

SUPPLEMENTARY FIGURES

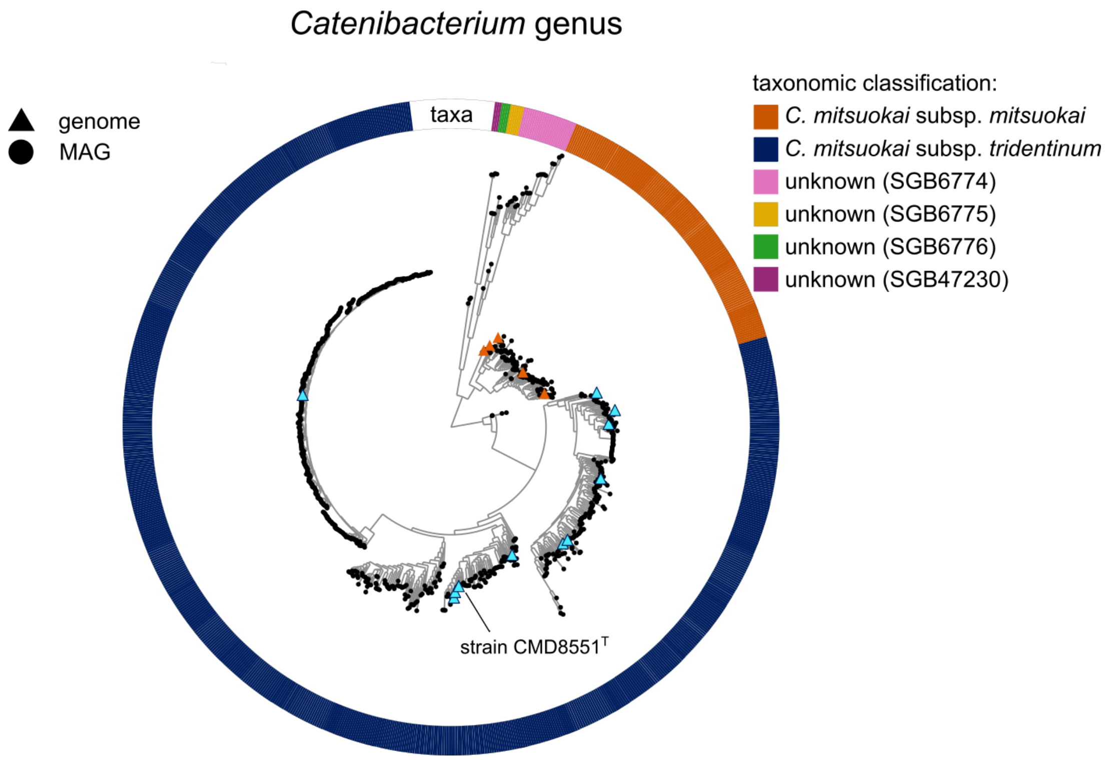

Figure S1. Phylogenetic tree of the *Catenibacterium* genus including all reference genomes as well as high quality MAGs (>90% completeness, <5% contamination) available in the ChocoPhlAnSGB database (Jan 25 version) (total n=1141). Only high-quality MAGs (>90% completeness, <5% contamination) were included in the analysis (n=865 genomes). Outer ring indicates species or subspecies-level taxonomic classification, with unknown taxa identified in the legend by Metaref SGB IDs.

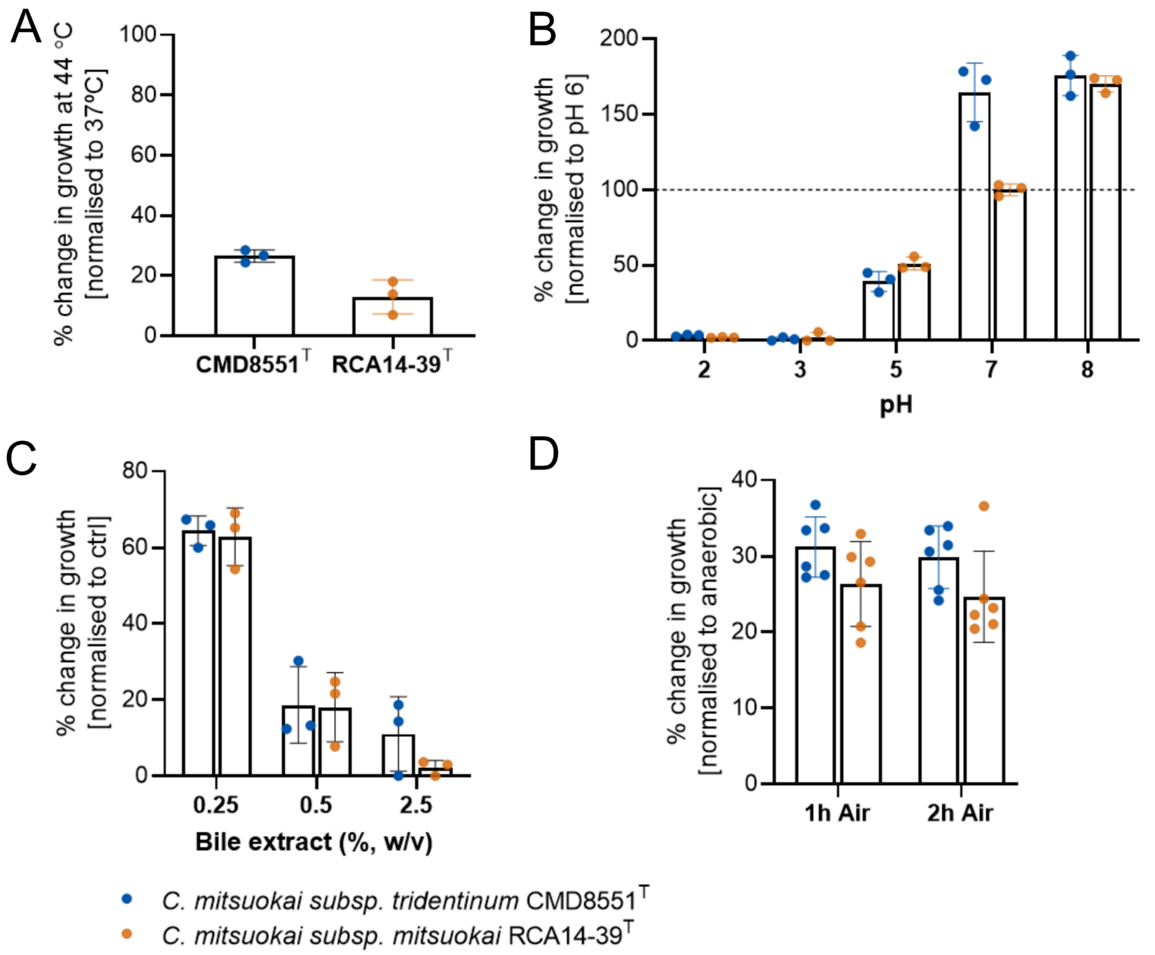

Figure S2. Scatter dot plot (mean ± SD) showing growth of *C. mitsuokai* subsp. *tridentinum* CMD8551^{T} and *C. mitsuokai* subsp. *mitsuokai* RCA14-39^{T} strains under environmental stressors: temperature (A), pH (B), bile acids (C), exposure to oxygen (D). Growth is represented as % change in optical density normalised to control.

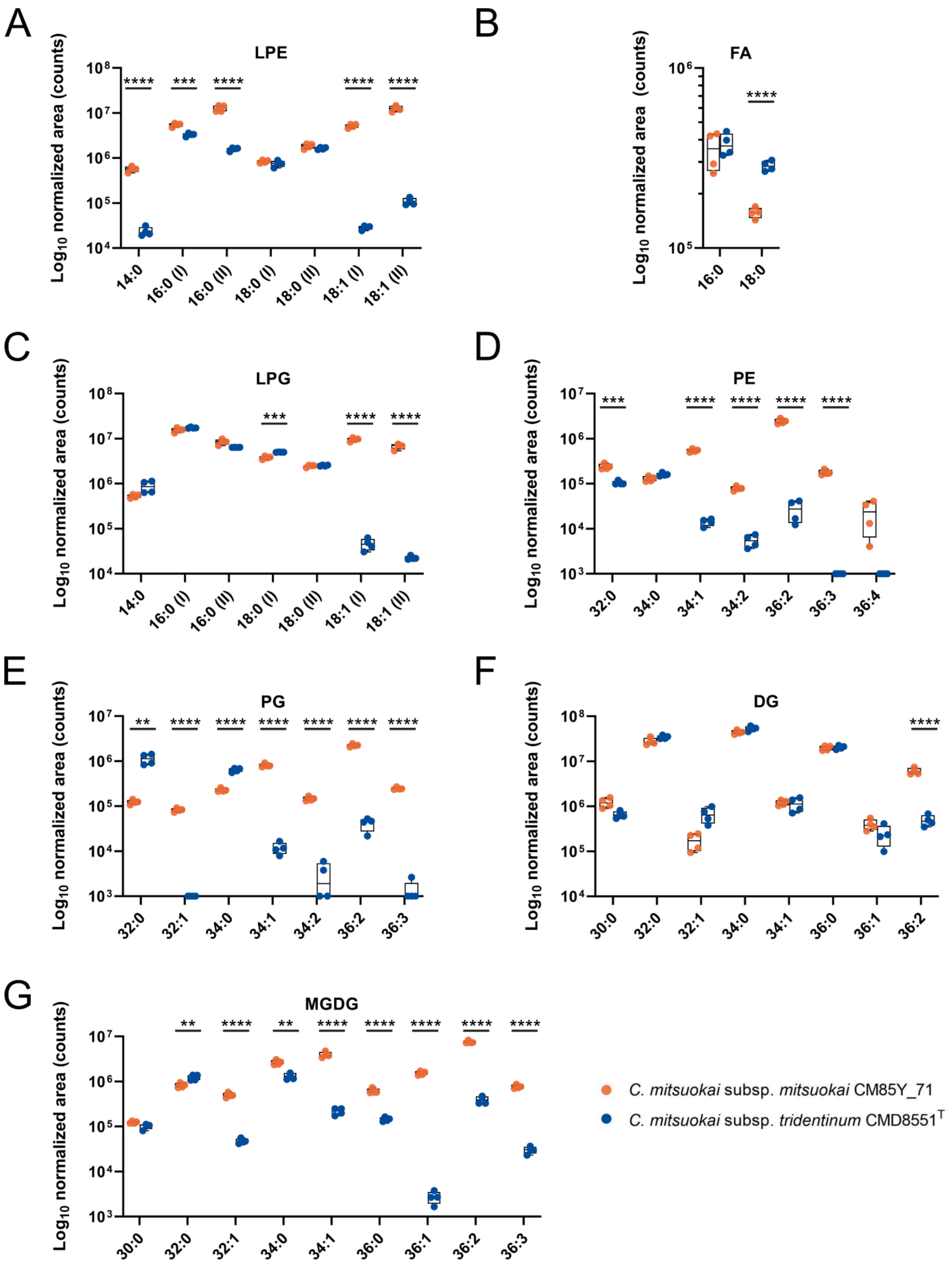
A
LPE
B
FA
C
LPG
D
PE
E
PG
F
DG
G
MGDG
Log10 normalized area (counts)
14:0
16:0 (I)
16:0 (II)
18:0 (I)
18:0 (II)
18:1 (I)
18:1 (II)
16:0
18:0
32:0
34:0
34:1
34:2
36:2
36:3
36:4
32:1
36:0
36:1
30:0
C. mitsuokai subsp. mitsuokai CM85Y_71
C. mitsuokai subsp. tridentinum CMD8551T

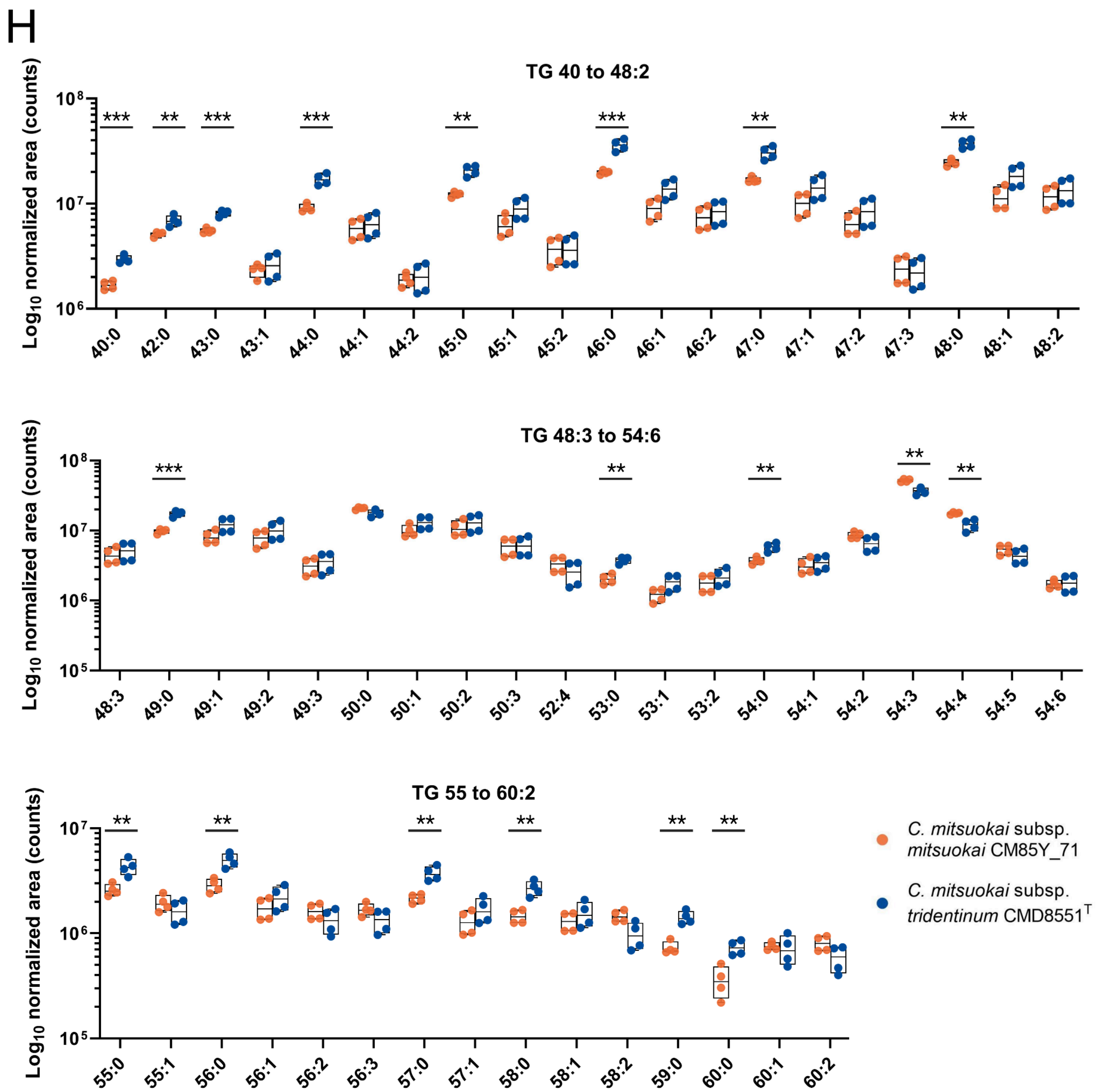

Figure S3. Boxplot (min to max) of chromatographic peak areas of single annotated lipidic compounds (divided by category, panel A to H) across *C. mitsuokai* subsp. *tridentinum* CMD8551^{T} and *C. mitsuokai* subsp. *mitsuokai* CM85Y_71. Each data point shows different technical replicates (n=4) injected for each biological sample. Peak areas were corrected for injection volume and sample concentration. Statistical significance asterisks refer to measurement noise, rather than biological variation, and were calculated via one unpaired t test per lipid class, without same SD assumption, followed by False Discovery Rate approach (Benjamini, Krieger and Yekutieli, with Q=1%) for *P* value adjustment (*q* value), *df*=6; $q \geq 0.05$, ns; $q < 0.05$, *; $q < 0.01$, **; $q < 0.001$, ***; $q < 0.0001$, ****. Abbreviations: LPE: lyso-phosphatidylethanolamines; FA: fatty acids; LPG: lyso-phosphatidylglycerols; PE: phosphatidylethanolamines; PG: phosphatidylglycerols; DG: diglycerides; MGDG: monogalactosyl-diacylglycerols; TG: triglycerides.
